# Comparative efficacy and toxicity of immune checkpoint inhibitors in combination with or without chemotherapy treatment for advanced esophageal squamous cell carcinoma: A systematic review and meta-analysis

**DOI:** 10.3389/fonc.2022.958783

**Published:** 2022-11-24

**Authors:** Yue Ma, Yu Xin, Dan Su, Yuxin Zhou, Hongxu Li, Haoyi Zou, Xuefan Yu, Qing Yang, Jie Cui, Changsong Wang, Yanqiao Zhang

**Affiliations:** ^1^Department of Gastrointestinal Medical Oncology, Harbin Medical University Cancer Hospital, Harbin, China; ^2^Department of Critical Care Medicine, Harbin Medical University Cancer Hospital, Harbin, China

**Keywords:** immune checkpoint inhibitors, esophageal squamous cell carcinoma, toxicity, efficacy, meta-analysis

## Abstract

**Introduction:**

We did a systematic review and meta-analysis to assess the efficacy and safety of immune checkpoint inhibitors combined with or without chemotherapies in patients with esophageal squamous cell carcinoma.

**Methods:**

Data related to the treatment of esophageal squamous cell carcinoma with immune checkpoint inhibitors therapy were retrieved from the database construction to August 2022. The risk of bias was assessed using the Cochrane Manual standard and RevMan 5.3 software for data synthesis. The outcome measures observed included overall survival, 12-month survival, disease control rate, objective response rate, treatment-related adverse events of grade 3 or higher, and progression-free survival. The adverse reactions included fatigue, diarrhea, hypothyroidism, rash, anemia, and anorexia.

**Results:**

In this meta-analysis, a total of 17 randomized controlled trials were included. In first-line therapy, immune checkpoint inhibitors combined with or without chemotherapy in the treatment of esophageal squamous cell carcinoma was more effective than chemotherapy alone. Overall survival, 12-month survival rate, and objective response rate were statistically significant. Among second-line treatments, immune checkpoint inhibitors combined with or without chemotherapy in the treatment of esophageal squamous cell carcinoma had statistically significant overall survival, 12-month survival, objective response rate, treatment-related adverse events of grade 3 or higher, and progression-free survival compared with chemotherapy alone.

**Conclusion:**

Both first- and second-line immune checkpoint inhibitors are effective for esophageal squamous cell carcinoma, and the adverse reactions are controllable and safe.

**Systematic review registration:**

https://www.crd.york.ac.uk/PROSPERO/, identifier CRD42021282586.

## 1 Introduction

Esophageal cancer ranks seventh in terms of incidence (604,000 new cases) and sixth in mortality overall (544,000 deaths); the latter means that esophageal cancer is responsible for 1 in every 18 cancer deaths in 2020. Approximately 70% of cases occur in men, and incidence and mortality rates are two- to threefold higher than in women (1). Esophageal cancer is one of the most important contribution of China to the worldwide burden of cancer, with epidemiological hot spots in Asia and Africa. In China, esophageal cancer is ranked as the third most common cancer in men and the fifth most common cancer in women and was the fourth leading cause of death from cancer in 2015 (2, 3). In addition, because of its anatomical location, esophageal cancer is often described as a disease. However, esophageal cancer has histological subtypes due to different etiologies; the most important subtypes include esophageal squamous cell carcinoma (ESCC) and esophageal adenocarcinoma (AC), of which esophageal squamous cell carcinoma accounts for 80%–90% (1, 4). Furthermore, the proportion of pathological types of esophageal cancer in China is significantly different from that in developed countries (5). In Western regions, nearly two-thirds of esophageal cancer cases are ACs (1). Therefore, a large number of clinical studies also pay attention to this issue when enrolling patients and often use organizational grade type or geographical location as stratification factors (6). This leads to different therapeutic outcomes based on histological subtypes.

Previous studies have shown that some factors play an important role in the occurrence and development of esophageal cancer. Gastroesophageal reflux disease and obesity are the main pathogenic factors of esophageal adenocarcinoma, while smoking and alcohol consumption are the main causes of esophageal squamous cell carcinoma (7). Gastroesophageal reflux disease and obesity-driven inflammation generate a pro-tumorigenic microenvironment consisting of pro-inflammatory M2-type macrophages, neutrophils, myeloid-derived suppressor cells (MDSCs) and TH2 cells, and proinflammatory mediators that include interleukin (IL)-1β, IL-8, IL-6, reactive oxygen species (ROS), and tumor-promoting TH2 cytokines. Because esophageal cancer TME is rich in immune cells, it is considered to be sensitive to ICIs (8).

One of the major breakthroughs in cancer treatment over the past decade has been the discovery of immune checkpoint proteins, which effectively suppress the immune system through a variety of mechanisms. Immune checkpoint inhibitors (ICIs) have revolutionized cancer treatment, showing higher efficacy in several cancers, such as non-small cell lung cancer (NSCLC), melanoma, malignant mesothelioma, renal cell carcinoma, urothelial carcinoma, gastric cancer, and head and neck carcinoma (9–16). When programmed cell death protein 1 (PD-1) or cytotoxic T-lymphocyte-associated protein 4 (CTLA-4) activates the immune checkpoint cascade, it leads to tumor-specific T-cell inactivation and immune evasion. Therapy with immune checkpoint inhibitors, such as anti-PD-1, anti-PD-L1, and/or anti-CTLA-4 drugs, can rejuvenate T cells and enable the adaptive immune system to target tumor cells (17–19).

As the biology of esophageal cancer might substantially vary in different regions of the world, so might the response to checkpoint inhibition (20). Recent studies concerning immunotherapeutic agents have been to revolutionize therapeutic strategies for esophageal cancer patients. Treatment with anti-PD-(L)1 drugs currently represents the mainstay of ESCC ICIs and can result in impressive response rates and durable disease remission, but only in a subset of patients.

Taking KEYNOTE-590 clinical study as an example, it examined first-line chemotherapy with or without pembrolizumab in patients with squamous cell carcinoma of the esophagus, adenocarcinoma of the esophagus, or Siewert-type GE junction adenocarcinoma. It demonstrated that pembrolizumab plus chemotherapy improved overall survival (OS) in patients with ESCC with PD-L1 CPS≥10 tumors, all squamous cell carcinomas, and all patients with CPS≥10. Progression-free survival (PFS) was also improved (21).

In this meta-analysis and literature review, we tried to analyze the efficacy and safety of ICIs combined with or without different chemotherapies in patients with ESCC.

## 2 Materials and methods

### 2.1 Literature search

We followed the Preferred Reporting Items for Systematic Reviews and Meta-analyses (PRISMA) guidelines for this systematic review and meta-analysis (22). We searched PubMed, The Cochrane Library, Embase, Web of Science, CNKI, Wanfang, VIP, and Biomedical database (from the establishment of the database to August 2022), by searching for articles on ICIs therapy with or without chemotherapy for ESCC. The method of combining mesh words and free words is used to search literatures. The language limit is Chinese and English. The search terms were “Esophageal Cancers,” “Neoplasm, Esophageal,” “programmed death receptor 1,” “Checkpoint Inhibitors, Immune,” “ocrelizumab,” “Nivolumab,” “pembrolizumab,” and “telimomab.” We also reviewed the references of articles included in the final selection. The detailed search strategy can be found in **Supplementary File 1**.

### 2.2 Study selection

Inclusion criteria were as follows: (1) studies whose subjects were patients with confirmed ESCC by pathological diagnosis; (2) studies that reported directly hazard ratios (HRs) and 95% confidence intervals (95%CIs) for OS or disease-free survival (DFS), or sufficient data were provided to calculate the HR and 95%CIs; (3) studies that included immune checkpoint inhibitor therapy for esophageal cancer; (4) studies whose target population was patients with advanced esophageal squamous cell carcinoma; and (5) abstracts and conference articles meeting the above inclusion criteria.

Excluded criteria were as follows: (1) meta-analyses, reviews, surveys, letters, case reports, and book chapters, and studies based on the National Cancer Database, or the Surveillance, Epidemiology, and End Results database; (2) duplicate publications; (3) single-arm clinical trial; (4) unable to obtain data; and (5) studies that have <10 cases.

### 2.3 Data extraction

Two authors independently extracted information from eligible articles. The authors resolved differences through discussion. The extracted data were as follows: (1) basic characteristics (first author, year of publication, study type, sample size, the protocol of test group and control group, and treatment line); (2) HRs and 95% CIs extracted for OS or DFS [if the studies did not directly report HRs and 95% CIs, we calculated the data based on the methods described by a previous study (23)]; and (3) the number of patients for each clinicopathological feature. If the relevant information of some items was not provided, these items were marked as “not available (NA).” When the HRs for the survival outcomes were reported by both univariate and multivariate analyses, only the HRs from the multivariate analysis were extracted (24).

### 2.4 Outcome

#### 2.4.1 The main outcome

The main outcomes were overall survival and objective response rate.

#### 2.4.2 The secondary outcome

Secondary outcomes were 12-month survival, disease control rate, treatment-related adverse events of grade 3 or higher, and progression-free survival.

### 2.5 Quality assessment of included studies

The quality of the included literatures was evaluated according to the standard Cochrane manual, including ① randomization method, ② distribution scheme hidden, ③ whether blind method is used, ④ integrity of result data, ⑤ selective reporting of research results, and ⑥ other sources of bias. The quality evaluation was carried out by two researchers independently and cross-checked. In case of disagreement, the decision was made by discussion or referring to the opinion of the third researcher.

The quality of the included literatures was evaluated according to the modified Jadad scale, including the following. ① Was the study described as randomized? ② Was the method of randomization appropriate, ③ Was the study described as blinded?④ Was the method of blinding appropriate? ⑤Was there a description of withdrawals and dropouts? ⑥ Was there a clear description of the inclusion/exclusion criteria? ⑦ Was the method used to assess adverse effects described? ⑧ Was the method of statistical analysis described? The quality evaluation was carried out by two researchers independently and cross-checked. In case of disagreement, the decision was made by discussion or referring to the opinion of the third researcher.

### 2.6 Statistical analysis

Meta-analysis was performed using RevMan 5.3 software provided by the Cochrane Collaboration. The count data were expressed by odds ratio OR and 95%CI. χ^2^ test was used for heterogeneity among included studies. The random-effect model was chosen if obvious heterogeneity was present (I^2^ > 50%); otherwise, the fixed-effect model was selected. If there was statistical heterogeneity among study results (p<0.1, I^2^>50%), the source of heterogeneity was analyzed. We used sensitivity analysis to determine the source of heterogeneity, and the factors that might lead to heterogeneity were subgroup analyzed.

## 3 Results

### 3.1 Literature retrieval results

**Figure 1:**
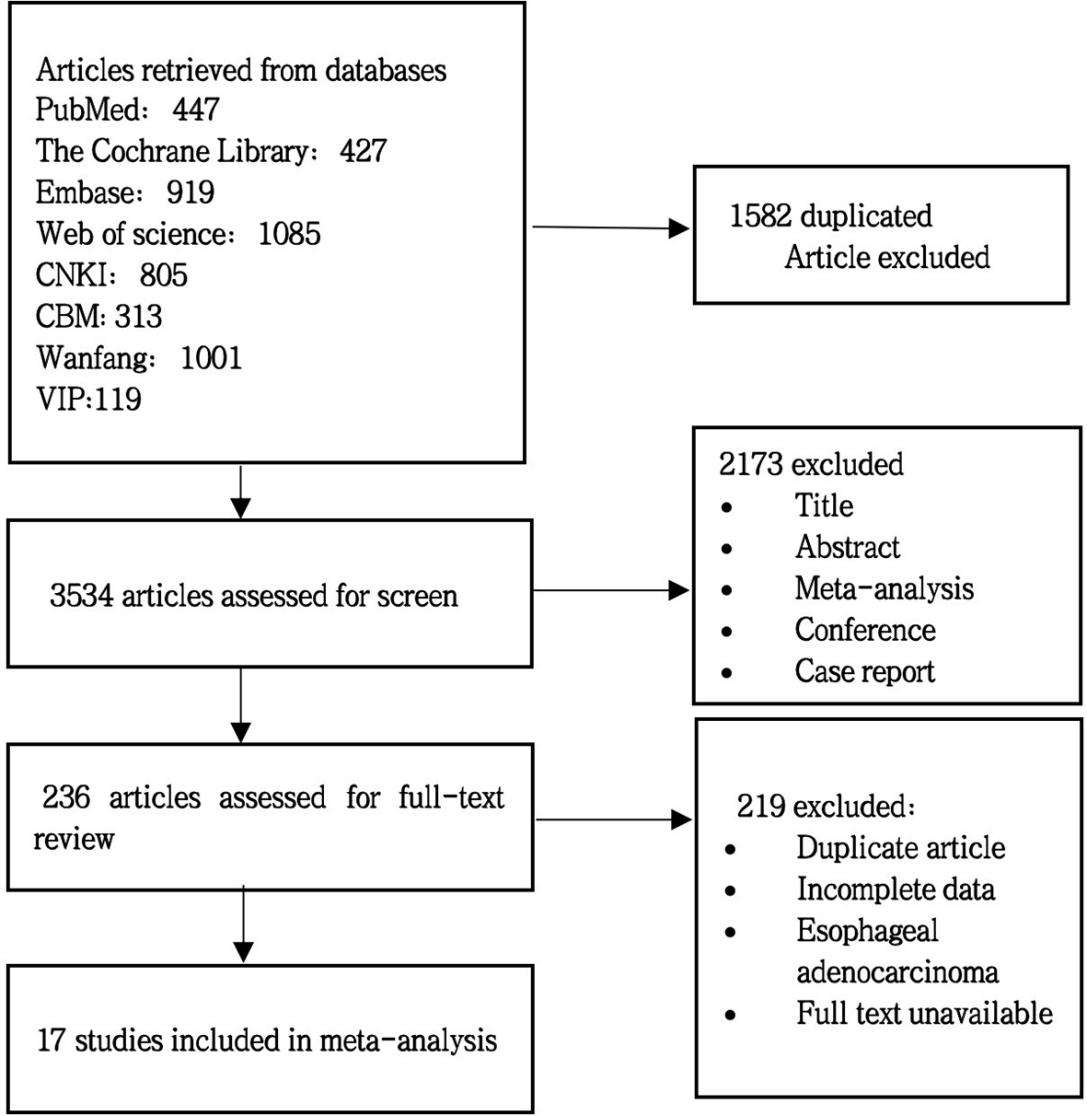
Literature screening flowchart.

A preliminary search was conducted for 5,116 articles, and 1,582 duplicate articles were deleted after review by literature management Endnote 20 software. A total of 3,534 articles inconsistent with the theme of this meta-analysis were excluded by reading the title and abstract. After full text screening, 17 randomized controlled trials were included. For specific screening process and results, see **Figure 1**.

### 3.2 Study characteristics

The 17 clinical trials included 8,080 patients (21, 25–37). Among them, 4,034 patients received immune checkpoint inhibitor treatment, and 4,046 patients did not receive the treatment. Eight studies were on first-line therapy; the rest were on second-line therapy. Seven studies reported adverse outcomes. The characteristics of each study are shown in **Table 1**.

**Table 1:** Baseline characteristics of studies included in the meta-analysis.

Author	Year	Country	People	Study design	Intervention	Control	pathology	ICIs	Intervention	Control	Treatment line	Outcomes	Adverse reaction
Huiyan Luo	2021	China	China	RCT	298	298	ESCC	PD-1	Camrelizumab+PTX+cis-platinum	Placebo+PTX+Cis-platinum	First line	ABCEFG	②④⑤⑥
Ian Chau	2021	NA	NA	RCT	321	324	ESCC	PD-1	Nivolumab+Chemotherapy	Fluorouracil+Cis-platinum	First line	ACD	NA
J. Ajani	2021	USA	various countries	RCT	256	256	ESCC	PD-1	Tislelizumab	PTX/Docetaxel/Irinotecan	Second line	F	NA
Jianming Xu	2020	China	China	RCT	95	95	ESCC	PD-1	Stintilimab	Chemotherapy	Second line	CDF	NA
Jing Huang	2020	China	China	RCT	228	220	ESCC	PD-1	Camrelizumab	Docetaxel/Irinotecan	Second line	ABCDEG	②③④⑥
Jong-Mu Sun	2021	Korea	various countries	RCT	274	274	ESCC	PD-1	Pembrolizumab+5-FU+Cis-platinum	Placebo+5-FU+Cis-platinum	First line	AG	NA
Ken Kato	2019	Japan	various countries	RCT	210	209	ESCC	PD-1	Nivolumab	Docetaxel/PTX	Second line	ABCEFG	①③④⑤⑥
Lin Shen	2021	China	China	RCT	327	332	ESCC	PD-1	Stintilimab+TP/CF	Placebo+TP/CF	First line	AC	NA
Masanobu Takahashi	2020	Japan	Japan	RCT	136	138	ESCC	PD-1	Nivolumab	PTX/Docetaxel	Second line	ACEG	①②③④⑤⑥
R.Xu	2021	China	China	RCT	257	257	ESCC	PD-1	Toripalimab+PTX+cis-platinum	Placebo+PTX+Cis-platinum	First line	BCDG	NA
Takashi Kojima	2020	Japan	various countries	RCT	198	203	ESCC	PD-1	Pembrolizumab	Docetaxel/PTX/Irinotecan	Second line	BCD	NA
Y.cao	2022	China	various countries	RCT	170	170	ESCC	PD-1	Pembrolizumab	PTX/Docetaxel/Irinotecan	First line	AEFG	NA
Xiaochuan Liu	2022	China	China	RCT	34	35	ESCC	PD-1	Sintilimab+PTX+5-FU+Cis-platinum	PTX+5-FU+Cis-platinum	Second line	AEFG	NA
Zhihao Lu	2022	China	various countries	RCT	327	332	ESCC	PD-1	Sintilimab+Chemotherapy	Placebo+Chemotherapy	Second line	NA	②③④⑤⑥
Lin Shen	2022	China	various countries	RCT	256	256	ESCC	PD-1	Tislelizumab	Chemotherapy	Second line	ABCDFG	①②③④⑥
H.Yoon	2022	Japan	various countries	RCT	326	323	ESCC	PD-1	Tislelizumab+Chemotherapy	Placebo+Chemotherapy	First line	DF	NA
Doki Y	2022	Japan	various countries	RCT	321	324	ESCC	PD-1	Nivolumab+Chemotherapy	Chemotherapy	First line	CDF	①②③④⑤⑥

Adverse reaction: ① Fatigue; ② Hypothyroidism; ③ Diarrhea; ④ Anemia; ⑤ Rash; ⑥ Decreased appetite; Outcomes: A,Overall survival; B,12-month overall survival; C,Treatment-related adverse reactions; D,Treatment-related adverse reactions of grade 3 or higher; E,Disease control rate; F,Objective response rate; G,progression-free survival; ESCC, esophageal squamous cell carcinoma; ICIs, Immune checkpoint inhibitors; NA,not available; PTX,paclitaxel; 5-FU,5-Fluorouracil.

### 3.3 Quality assessment

**Figure 2:**
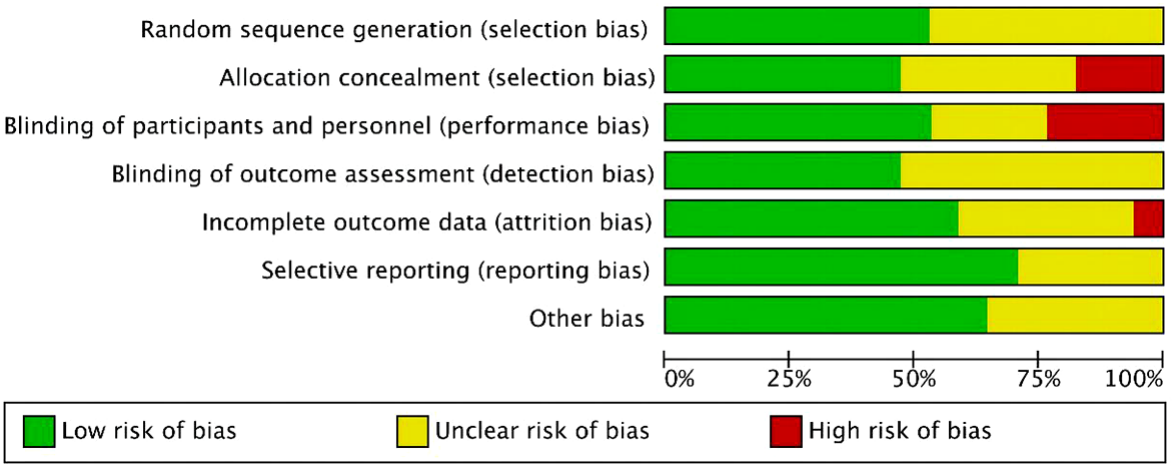
Risk of bias and applicability concerns summary.

**Figure 3:**
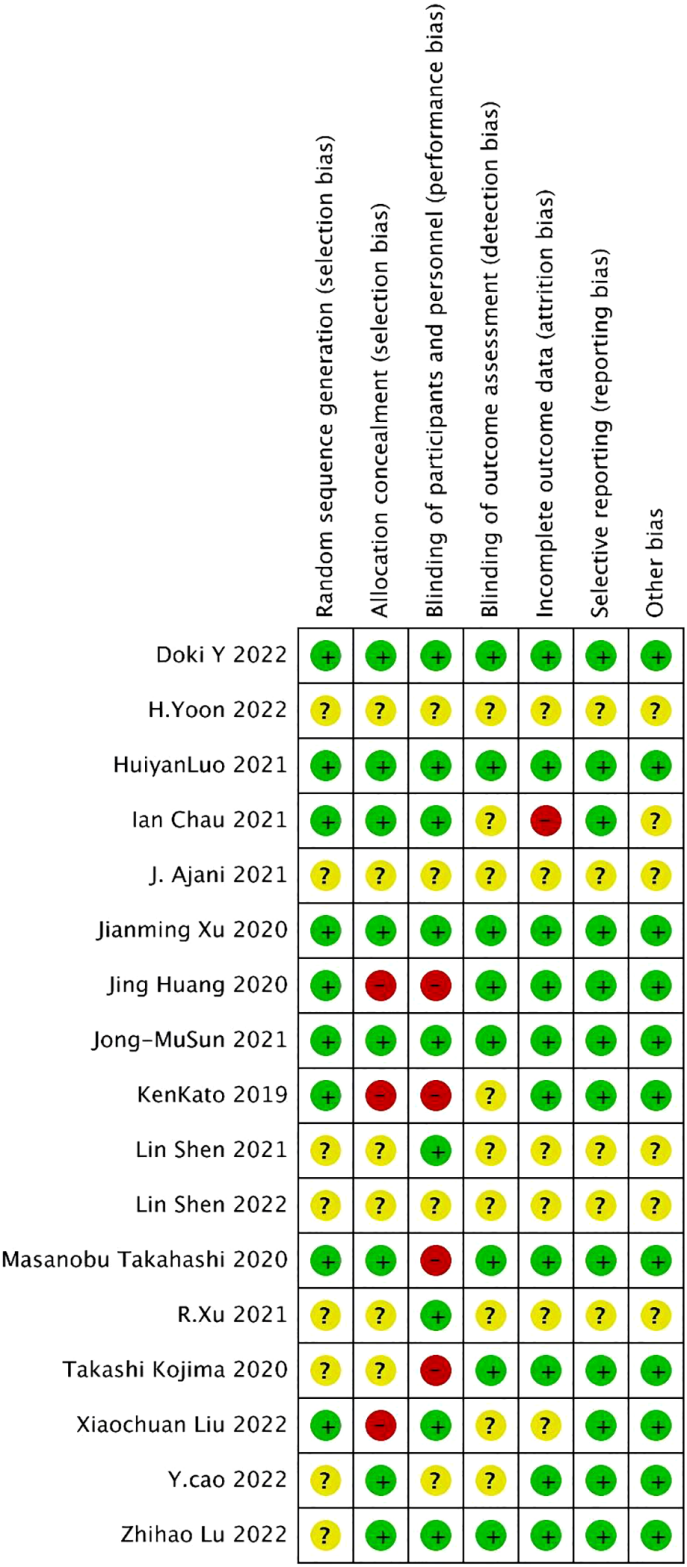
Risk of bias and applicability concerns graph.

For the 17 included literatures, Review Manager’s own literature evaluation tool was used for evaluation. Four literatures were conferences or abstracts, and the full text could not be obtained. We obtained the methodological part of relevant trials from the clinical trial registration website (www.clinicaltrials.gov/) for quality evaluation (**Figures 2**, **3**).

### 3.4 Meta-analysis

In first-line therapy, immune checkpoint inhibitors combined with or without chemotherapy in the treatment of esophageal squamous cell carcinoma was more effective than chemotherapy alone. Overall survival, 12-month survival rate, and objective response rate were statistically significant. Among second-line therapy, immune checkpoint inhibitors combined with or without chemotherapy in the treatment of esophageal squamous cell carcinoma had statistically significant overall survival, 12-month survival, objective response rate, treatment-related adverse events of grade 3 or higher, and progression-free survival compared with chemotherapy alone. In the adverse effects, hypothyroidism and decreased appetites have statistical significance.

#### 3.4.1 First-line ICIs

##### 3.4.1.1 Overall survival

**Figure 4:**
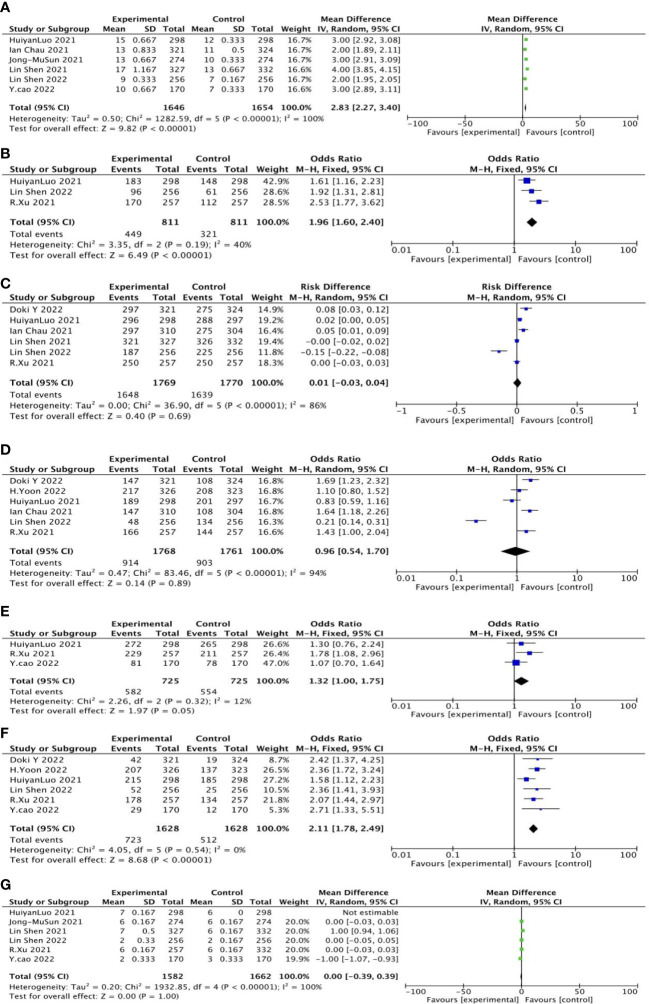
General forest plot for each outcome in first-line treatment.

OS was reported in six studies, including 1,646 cases in the experimental group and 1,654 cases in the control group. The results of heterogeneity analysis were p<0.00001, I^2 =^ 100%, indicating great heterogeneity. Therefore, the random effect model was adopted. The results of the meta-analysis showed that OR and 95%CI=2.83[2.27, 3.40], p<0.00001, p< 0.05, suggesting that the difference in overall survival rate between the two groups was statistically significant (**Figure 4A**).

##### 3.4.1.2 Twelve-month overall survival

The 12-month overall survival rate was reported in three studies, including 811 patients in the experimental group and 811 cases in the control group. The results of heterogeneity analysis were p=0.19 and I^2 =^ 40%, indicating small heterogeneity. Therefore, the fixed effect model was adopted. Meta-analysis results showed that OR and 95%CI=1.96[1.60, 2.40], p<0.0001, p<0.05, suggesting that the 12-month overall survival rate between the two groups was statistically significant (**Figure 4B**).

##### 3.4.1.3 Treatment-related adverse reactions

Six studies reported treatment-related adverse reactions, including 1,769 cases in the experimental group and 811 cases in the control group. The results of heterogeneity analysis were p<0.00001, I^2 =^ 86%, indicating great heterogeneity. Therefore, the random effect model was adopted. Meta-analysis results showed that OR and 95%CI=0.01[−0.03, 0.04], p=0.69, p>0.05, suggesting that there was no significant difference in treatment-related adverse reactions between the two groups (**Figure 4C**).

##### 3.4.1.4 Treatment-related adverse reactions of grade 3 or higher

Six studies reported treatment-related adverse reactions of more than grade 3, including 1,768 cases in the experimental group and 1,716 cases in the control group. The results of heterogeneity analysis were p<0.00001, I^2 =^ 94%, indicating great heterogeneity. Therefore, the random effect model was adopted. Meta-analysis results showed that OR and 95%CI=0.96[0.54, 1.70], p=0.89, p>0.05, suggesting that there was no significant difference between the two treatment-related adverse reactions of grade 3 or higher (**Figure 4D**).

##### 3.4.1.5 Disease control rate

The disease control rate was reported in three studies, including 725 cases in the experimental group and 725 cases in the control group. The results of heterogeneity analysis were p=0.32, I^2 =^ 12%, indicating small heterogeneity. Therefore, the fixed effect model was adopted. Meta-analysis results showed that OR and 95%CI=1.32[1.00, 1.75], p=0.05, p≥0.05, suggesting that there was no significant difference in disease control rates between the two groups (**Figure 4E**).

##### 3.4.1.6 Objective response rate

Objective response rate was reported in six studies, including 1,628 cases in the experimental group and 1,628 cases in the control group. The results of heterogeneity analysis were p=0.54, I^2 =^ 0%, indicating small heterogeneity. Therefore, the fixed effect model was adopted. Meta-analysis results showed that OR and 95%CI=2.11[1.78, 2.49], p<0.00001, p<0.05, suggesting that there was a statistically significant difference in objective response rate between the two groups (**Figure 4F**).

##### 3.4.1.7 Progression-free survival

PFS was reported in six studies, including 1,582 cases in the experimental group and 1,662 cases in the control group. The results of heterogeneity analysis were p< 0.00001, I^2 =^ 100%, indicating great heterogeneity. Therefore, the random effect model was adopted. Meta-analysis results showed that OR and 95%CI=0.00[−0.39, 0.39], p=1, P>0.05, suggesting that there was no significant difference in PFS between the two groups (**Figure 4G**).

#### 3.4.2 Second-line treatment

##### 3.4.2.1 Overall survival

**Figure 5:**
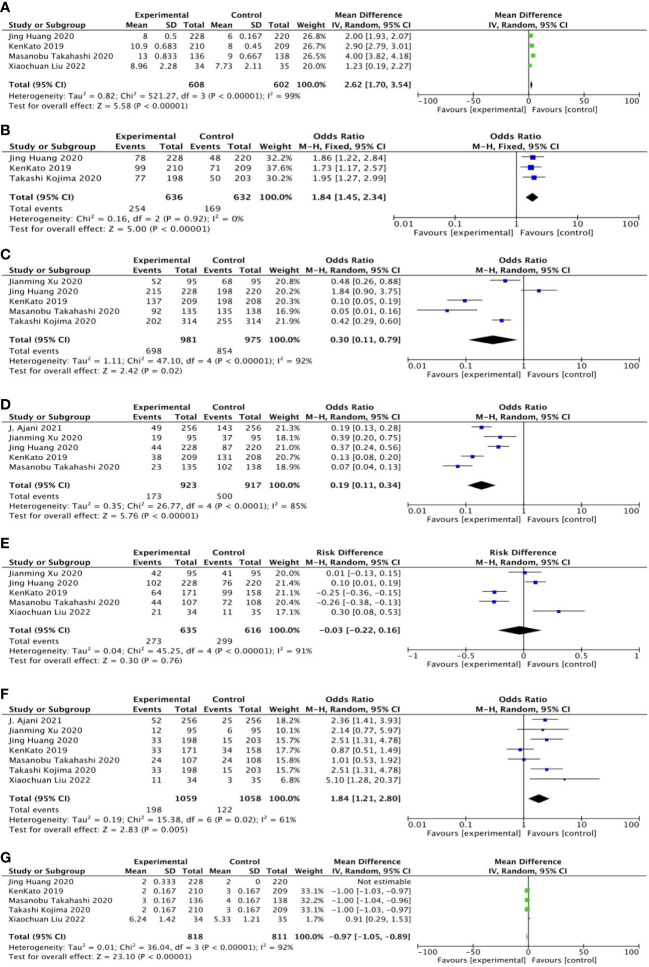
General forest plot for each outcome in second-line treatment.

OS was reported in four studies, including 608 cases in the experimental group and 602 cases in the control group. The results of heterogeneity analysis were p < 0.00001, I^2 =^ 99%, indicating great heterogeneity. Therefore, the random effect model was adopted. The results of meta-analysis showed that OR and 95%CI=2.62[1.70, 3.54], p < 0.00001, p<0.05, suggesting that the OS between the two groups was statistically significant (**Figure 5A**).

##### 3.4.2.2 Twelve-month overall survival

Twelve-month overall survival was reported in three studies, including 636 patients in the experimental group and 632 cases in the control group. The results of heterogeneity analysis were as follows: p=0.92, I2 = 0%, indicating small heterogeneity. Therefore, the fixed effect model was adopted. Meta-analysis results showed that OR and 95%CI=1.84[1.45, 2.34], p<0.00001, p < 0.05, suggesting that there was significant difference in 12-month overall survival rate between the two groups (**Figure 5B**).

##### 3.4.2.3 Treatment-related adverse reactions

Five studies reported treatment-related adverse reactions, including 981 cases in the experimental group; the control group included 975 cases. The results of heterogeneity analysis were as follows: p < 0.00001, I^2 =^ 92%, indicating great heterogeneity. Therefore, the random effect model was adopted. The results of meta-analysis showed that OR and 95%CI=0.30[0.11, 0.79], p=0.02, p < 0.05, suggesting that there was statistically significant difference in treatment-related adverse reactions between the two groups (**Figure 5C**).

##### 3.4.2.4 Treatment-related adverse reactions of grade 3 or higher

Five studies reported treatment-related adverse reactions of grade 3 or higher, including 923 cases in the experimental group; the control group included 917 cases. The results of heterogeneity analysis were as follows: p < 0.0001, I^2 =^ 85%, indicating great heterogeneity. Therefore, the random effect model was adopted. The results of meta-analysis showed that OR and 95%CI=0.19[0.11, 0.34], p<0.00001, p < 0.05, suggesting that the difference in treatment-related adverse reactions above grade 3 between the two groups was statistically significant (**Figure 5D**).

##### 3.4.2.5 Disease control rate

Disease control rates were reported in five studies, including 635 patients in the experimental group; the control group included 616 cases. The results of heterogeneity analysis were as follows: p < 0.00001, I^2 =^ 91%, indicating great heterogeneity. Therefore, the random effect model was adopted. Meta-analysis results showed that OR and 95%CI=−0.03[−0.22,0.16], p=0.76, p > 0.05, indicating no statistically significant difference in disease control rates between the two groups (**Figure 5E**).

##### 3.4.2.6 Objective response rate

Objective response rates were reported in seven studies, including 1,059 patients in the experimental group and 1,058 cases in the control group. The results of heterogeneity analysis were as follows: p=0.02, I2 = 61%, indicating great heterogeneity. Therefore, the random effect model was adopted. Meta-analysis results showed that OR and 95%CI=1.84[1.21, 2.80], p=0.005, p < 0.05, suggesting that there was statistical significance in the difference in objective response rate between the two groups (**Figure 5F**).

##### 3.4.2.7 Progression-free survival

PFS was reported in five studies, including 818 cases in the experimental group; the control group included 811 patients. The results of heterogeneity analysis were as follows: p < 0.00001, I^2 =^ 92%, indicating great heterogeneity. Therefore, the random effect model was adopted. The results of meta-analysis showed that OR and 95%CI=−0.97[−1.05, −0.89], p<0.00001, p<0.05, suggesting that the difference in PFS between the two groups was statistically significant (**Figure 5G**).

#### 3.4.3 Adverse reactions

##### 3.4.3.1 Weakness

**Figure 6:**
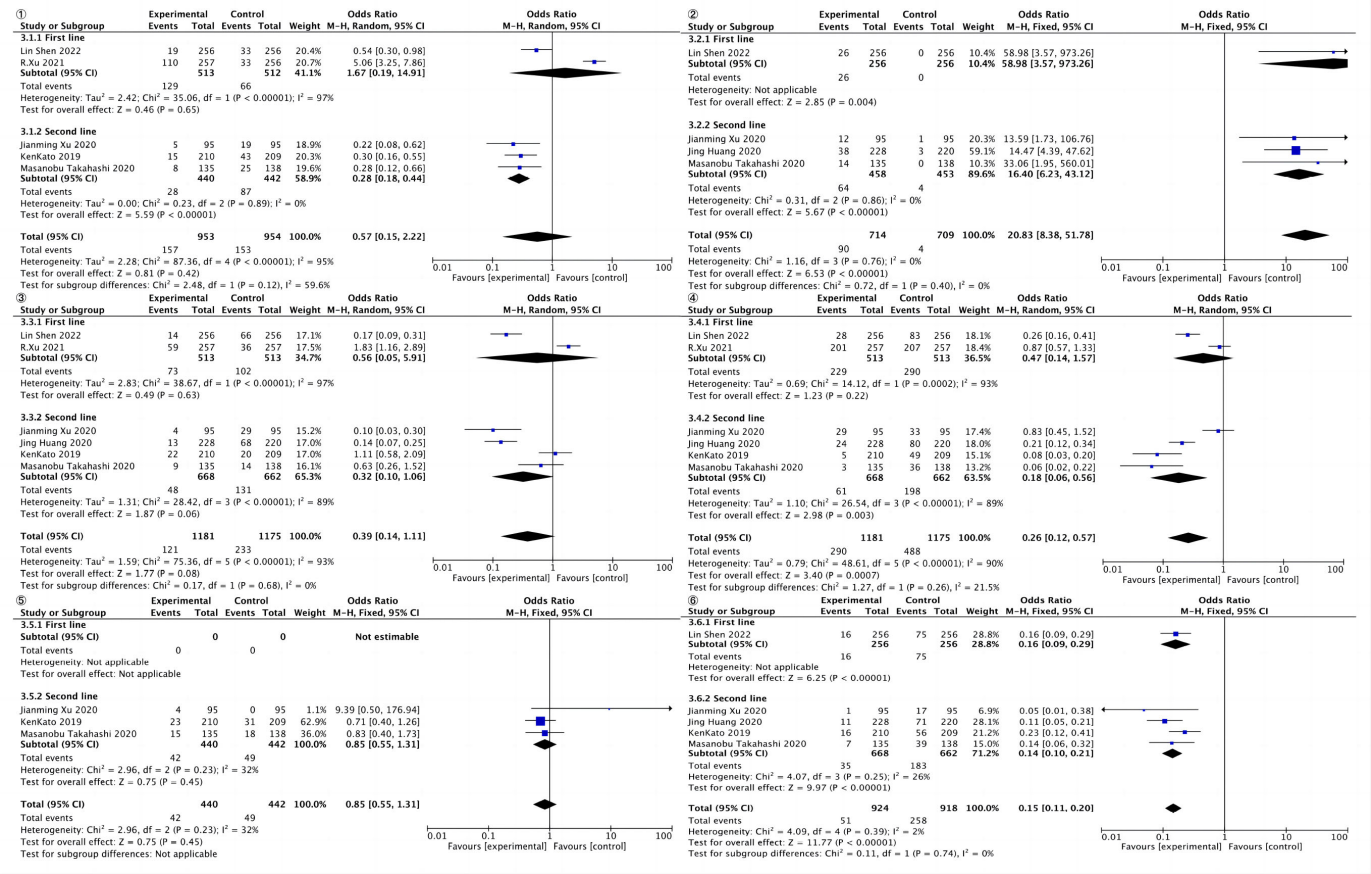
Total forest of adverse reactions.

Weakness was reported in five studies, including 953 cases in the experimental group; the control group included 954 cases. The results of heterogeneity analysis were p<0.00001, I^2 =^ 95%, indicating great heterogeneity. Therefore, the random effect model was adopted. The results of meta-analysis showed that OR and 95%CI=0.57[0.15, 2.22], p=0.42, p>0.05, suggesting no statistically significant difference in weakness between the two groups. (**Figure 6**, ①)

##### 3.4.3.2 Hypothyroidism

Hypothyroidism was reported in four studies, including 714 cases in the experimental group and 709 cases in the control group. The results of heterogeneity analysis were p=0.76 and I^2 =^ 0%, indicating small heterogeneity. Therefore, the fixed effect model was adopted. The results of meta-analysis showed that OR and 95%CI=20.83[8.38, 51.78], p<0.00001, p<0.05, suggesting that there was statistically significant difference in hypothyroidism between the two groups. (**Figure 6**, ②)

##### 3.4.3.3 Diarrhea

Diarrhea was reported in six studies, including 1,181 cases in the experimental group and 1,175 cases in control group. The results of heterogeneity analysis were p < 0.0001, I^2 =^ 93%, indicating great heterogeneity. Therefore, the random effect model was adopted. Meta-analysis results showed that OR and 95%CI=0.39[0.14, 1.11], p=0.08, p>0.05, indicating no statistically significant difference in diarrhea between the two groups (**Figure 6**, ③).

##### 3.4.3.4 Anemia

Anemia was reported in six studies, including 1,181 cases in the experimental group; the control group included 1,175 cases. The results of heterogeneity analysis were p<0.00001, I^2 =^ 90%, indicating great heterogeneity, so the random effect model was adopted. The results of meta-analysis showed that OR and 95%CI=0.26[0.12, 0.57], p=0.0007, p<0.05, suggesting statistically significant difference in anemia between the two groups (**Figure 6**, ④).

##### 3.4.3.5 Rash

Rashes were reported in three studies, including 440 cases in the experimental group; the control group included 442 cases. The results of heterogeneity analysis were p=0.23 and I^2 =^ 32%, indicating great heterogeneity. Therefore, the random effect model was adopted. Meta-analysis results showed that OR and 95%CI=0.85[0.55, 1.31], p=0.45, p>0.05, indicating no statistically significant difference in rash between the two groups (**Figure 6**, ⑤).

##### 3.4.3.6 Decreased appetites

Decreased appetites were reported in five studies, including 924 cases in the experimental group; the control group contained 918 patients. The results of heterogeneity analysis were as follows: p=0.39, I^2 =^ 2%, indicating small heterogeneity. Therefore, the fixed effect model was adopted. The results of meta-analysis showed that OR and 95%CI=0.15[0.11, 0.20], p<0.00001, p<0.05, suggesting statistically significant difference in decreased appetites between the two groups (**Figure 6**, ⑥).

## 4 Discussion

In recent years, the randomized exploration of ICIs treatments has drawn extensive attention in many patient categories with promising results, and esophageal cancer had entered the era of ICIs.

However, in patients with advanced esophageal squamous cell carcinoma, the efficacy of immune checkpoint inhibitors is not known, so we performed meta-analysis, and we also performed meta-analysis of related adverse effects. In 2019, KEYNOTE-181 showed that second-line treatment with PD-L1-positive advanced/metastatic esophageal cancer or esophagogastric junction adenocarcinoma with PD-L1 monotherapy significantly extended patients’ overall survival (OS) compared to standard chemotherapy (25). Subsequently, similar results were observed in the ESCORT study, Attract-03 study, and RATIONALE-302. Since then, ICIs have become the standard second-line treatment for patients with ESCC (26–28). Therefore, in the indication of second-line treatments, an enrichment strategy design led to the approval of ICIs for PD-L1-high patients. Our study shows that among second-line treatments, ICIs combined with chemotherapy in the treatment of esophageal squamous cell carcinoma had statistically significant OS, 12-month overall survival (12 OS), treatment-related adverse reactions, treatment-related grade 3 or more adverse reactions, objective response rate (ORR), and PFS compared with chemotherapy alone.

As the first-line clinical trials of advanced esophageal cancer data published, ICIs have become the standard of treatment of first-line esophageal cancer. ICIs combined with chemotherapy in the first-line treatment of esophageal cancer ameliorate the clinical outcomes and improve survival benefits in patients with ESCC. At the same time, the ORR increased from 27%–45% in the chemotherapy group to 45%–72.1% with the addition of ICIs.

Based on the results of our meta-analysis of esophagogastric cancer chemotherapy regimens with or without ICIs, a total of 17 RCTs were included in this meta-analysis, involving a total of 8,080 patients with esophageal cancer; the major conclusions can be drawn that may directly affect clinical practice. In KEYNOTE-590 study (21) and CHECKMATE 648 study (29), cisplatin +5-FU (FP) regimen was selected in the control group, and the overall ORR rate of patients was <30%. The ORR rate of Taxol + cisplatin (TP) was 45% in the ESCORT-1 (30), Orient-15 (31), and Jupit-06 (32) studies. Although not a head-to-head clinical study, a significant difference in effectiveness between FP and TP regimens was observed. Relevant studies have confirmed that in synchronous chemoradiotherapy for patients with esophageal cancer, there is no significant difference in the efficacy of different chemotherapy schemes combined with radiotherapy. However, the prognosis of ICIs combined with different chemotherapy regimens is different. This may be related to the synergistic effect of ICIs combined with chemotherapy. A previous network meta-analysis showed that the efficacy and safety of different first-line chemotherapy regimens for esophageal cancer were different (38). At the same time, it also reflects that the synergistic effect of different chemotherapy regimens on ICIs is also different. Some studies indicate that 5-Fu/DDP could induce immunogenic cell death in the tumor microenvironment of ESCC. However, the interaction between ICIs and chemotherapy is still unknown. They showed that immunogenic cell death (ICD) was induced in ESCC by proving the maturation of DCs (39). As a consequence, compared with FP regimen, combining with TP regimen is more effective, which provides a basis for the selection of chemotherapy regimen.

Our results show that, in the first-line treatment of esophageal squamous cell carcinoma, ICIs combined with chemotherapy was more effective than chemotherapy alone. OS, 12-month survival rate, and objective response rate were statistically significant. ICIs antitumor mechanisms lead to the particularity of its adverse effects; the effects on the body’s immune system will produce the corresponding adverse reaction, In our meta-analysis, in the treatment with ICIs alone compared with chemotherapy alone in the second-line treatment, related adverse reactions and incidence of grade ≥3 TRAEs are statistically significant, but significant statistical differences were not observed in the first-line treatment. Compared to traditional chemotherapy, ICIs have generally fewer side effects.

In addition, six of the most common ICI-related adverse reactions were analyzed. It was found that ICIs often resulted in hypothyroidism, anemia, and loss of appetite. The toxic and side effects of chemotherapy drugs mainly include cytopenia (white blood cells, platelets, hemoglobin, granulocytes, etc.), gastrointestinal reactions (nausea, vomiting, diarrhea, and constipation), liver and kidney dysfunction, cardiotoxicity, and nerve dysfunction (40). Our results indicate that ICIs have more advantages in adverse reactions than chemotherapy, but drug reactions should be carefully monitored and treated in time during drug administration.

Previous studies have demonstrated that there are many risk factors for irAEs, for example, sarcopenia, sex, tumor histology, underlying comorbidities, treatment modality, concurrent medications, preexisting autoantibodies, cytokine assays (IL-6, IL-10, IL-17, CXCL9, CXCL10, CXCL11 etc.), blood cells, gut microbiome, and genetic variability (41).This may explain why adverse effects were not statistically significant in the ICIs group compared with the control group in the first-line treatment of ESCC. However, when esophageal cancer patients are treated with second-line therapy, their nutritional status is often poor, and their skeletal muscle is decreased, which may lead to severe immune-related adverse reactions. Therefore, compared with the chemotherapy alone group, the adverse reactions of ICIs are significantly more serious.

Currently, PD-L1 is a biomarker that can benefit from ICIs. In KEYNOTE-590 study and CHECKMATE 648 study, patients with positive PD-L1 expression had an even greater reduction in risk of death. In the subgroup of patients with negative PD-L1 expression, the risk of death was not significantly reduced, and none of these patients could benefit from combination therapy (21, 29). However, in the ESCORT 1 study, Orient-15 study, and Jupit-06 study led by Chinese experts, the expression of PD-L1 seemed to have little influence on the final benefit of patients after the combination of ICIs and chemotherapy (30–32). The reason may be that TP and FP chemotherapy regimens commonly used in Chinese patients are more effective than those in relevant studies in western countries, resulting in higher benefits for the whole population. In addition, ethnic differences between eastern and western patients lead to different responses to ICIs and different efficacy. Therefore, the detection of PD-L1 expression is still necessary and is the best predictor of efficacy at present. However, it is still necessary to consider the related effects of different race and chemotherapy regimen, combined with the clinical characteristics of patients and other relevant markers, to more accurately identify the beneficiaries of ICIs.

In the field of treatment of esophageal squamous cell carcinoma, the exploration of biomarkers is very important. Biomarkers also need to be combined with clinical features related to esophageal cancer. At the same time, the representativeness of PD-L1 expression results is still limited due to the small size of esophageal cancer biopsy samples. In the future, it is an important research trend to explore more representative biomarkers.

Although ICIs have shown some benefit in ESCC, because of both intrinsic and acquired immune resistance, there are still a large number of patients who do not respond well to ICIs. Research has shown comprehensively characterized tumor-infiltrating immune cells and revealed the landscape of the suppressive immune state for ESCC. In the ESCC TME, there were exhausted T cells, exhausted NK cells, regulatory T (Treg) cells, alternatively activated macrophages (M2), and tolerogenic dendritic cells (tDCs) in these tumors, indicating an inflamed but immune-suppressed TME in ESCC (42).

Some limitations of this work should be acknowledged. Meta-analysis is inherently observational, and, despite our best efforts to investigate inconsistency and to assess the impact of effect modifiers using sensitivity analysis, it is possible that the results are affected by unmeasured confounding. Estimates that rely substantially on indirect evidence should be interpreted with care. In addition, since many large RCTS are currently under way, this paper includes the conferences and abstracts with available data at present, which is highly likely to produce bias in some literatures. Due to the limited literature included for each outcome index, it is impossible to analyze the source of the variance. The dose of immunosuppressant is different from the regimen, and there is no unified standard. Of course, this paper also has certain advantages, for example, the study subjects were all patients with esophageal squamous cell carcinoma, and more outcome indicators and adverse reactions were collected. The era of ICIs for esophageal squamous cell carcinoma has arrived, and the future is promising. However, ICIs in the field of ESCC has a long way to go.

## Data availability statement

The original contributions presented in the study are included in the article/**Supplementary Material**. Further inquiries can be directed to the corresponding authors.

## Author contributions

YM, YX, and DS wrote the manuscript. YXZ, HL, and HZ performed the literature review. XY, QY, and JC performed the statistical analysis. CW and YQZ revised the text. All authors read and approved the final manuscript.

## Funding

This work was supported by the grants from the National Natural Science Foundation of China No. 82173233.

## Conflict of interest

The authors declare that the research was conducted in the absence of any commercial or financial relationships that could be construed as a potential conflict of interest.

## Publisher’s note

All claims expressed in this article are solely those of the authors and do not necessarily represent those of their affiliated organizations, or those of the publisher, the editors and the reviewers. Any product that may be evaluated in this article, or claim that may be made by its manufacturer, is not guaranteed or endorsed by the publisher.

## Supplementary material

Supplement File 1 Detailed retrieval strategy and PICOs.

Supplement File 2 modified Jadad scale.

Supplement File 3 Sensitivity analysis.

## Abbreviations

ICIs, immune checkpoint inhibitors; OS, overall survival; DCR, disease control rate; ORR, objective response rate; PFS, progression-free survival; RCTs, randomized controlled trials; ESCC, esophageal squamous cell carcinoma; AC, esophageal adenocarcinoma; PD-1, programmed cell death protein 1; PD-L1, programmed death ligand 1; CTLA-4, cytotoxic T-lymphocyte-associated protein 4; IL, interleukin; ROS, reactive oxygen species; MDSCs, myeloid-derived suppressor cells; HRs, hazard ratios; CI, confidence intervals
