# Supplementary material for: Comparative efficacy and toxicity of immune checkpoint inhibitors in combination with or without chemotherapy treatment for advanced esophageal squamous cell carcinoma: A systematic review and meta-analysis
Source: Front Oncol. 2022 Nov 24;12:958783. doi: 10.3389/fonc.2022.958783 (PMC9748809; doi:10.3389/fonc.2022.958783)
Supplement: Supplement File 1 — Detailed retrieval strategy and PICOs. [file Table_1.docx]

**PICOS model**

| P(Population ) | I(Intervention) | C(Comparison) | O(Outcomes) | S(Study design) | Combining  search terms |
| --- | --- | --- | --- | --- | --- |
| - Esophageal Neoplasms - esophagus tumor - Neoplasm, Esophageal - Esophagus Neoplasm - Esophagus Neoplasms - Neoplasm, Esophagus - Neoplasms, Esophagus - Neoplasms, Esophageal - Cancer of Esophagus - Cancer of the Esophagus - Esophagus Cancer - Cancer, Esophagus - Cancers, Esophagus - Esophagus Cancers - Esophageal Cancer - Cancer, Esophageal - Cancers, Esophageal - Esophageal Cancers | - [Immune Checkpoint Inhibitors](https://pubmed2.ilibs.cn/mesh/2051964) - Checkpoint Inhibitors, Immune - Immune Checkpoint Inhibitor - Checkpoint Inhibitor, Immune - Immune Checkpoint Blockers - Checkpoint Blockers, Immune - Immune Checkpoint Blockade - Checkpoint Blockade, Immune - Immune Checkpoint Inhibition - Checkpoint Inhibition, Immune - PD-L1 Inhibitor - Programmed Death-Ligand 1 Inhibitors - Programmed Death Ligand 1 Inhibitors - CTLA 4 Inhibitor - Cytotoxic T-Lymphocyte-Associated Protein 4 Inhibitors - Cytotoxic T Lymphocyte Associated Protein 4 Inhibitors - Cytotoxic T-Lymphocyte-Associated Protein 4 Inhibitor - Cytotoxic T Lymphocyte Associated Protein 4 Inhibitor - PD-1 Inhibitors - PD 1 Inhibitors - PD-1 Inhibitor - Inhibitor, PD-1 - PD 1 Inhibitor - Programmed Cell Death Protein 1 Inhibitor - Programmed Cell Death Protein 1 Inhibitors - PD-1-PD-L1 Blockade - Blockade, PD-1-PD-L1 - PD 1 PD L1 Blockade - PD-1 Protein - PD 1 Protein - PD-1 Receptor - PD 1 Receptor - Receptor, PD-1 - Antigens, CD279 - CD279 Antigens - CD279 Antigen - Antigen, CD279 - PD1 Receptor - Receptor, PD1 - Programmed Cell Death Protein 1 - Programmed Cell Death 1 Protein - carrelizumab - SHR-1210 - Opdivo - ONO-4538 - MDX-1106 - BMS-936558 - SCH-900475 - Keytruda - MK-3475 - lambrolizumab - tislelizumab - toripalimab - BGB-A317 - Nivolumab - pembrolizumab - keytruda - atezolizumab - tecentriq - imfinzi - durvalumab - SHR-1210 - camrelizumab - bavencio - avelumab - toripalimab - cemiplimab | - Chemotherapy | - overall survival (OS) - 12-month survival - disease control rate - objective response rate - treatment-related adverse events of grade 3 or higher - progression-free survival - Fatigue - Diarrhea - Hypothyroidism - Rash - anemia - anorexia | - Randomized controlled trial - RCT - randomized controlled - clinical trial | P and I and S |

**1.SourcesEmbase, MEDLINE :919**

Query('esophagus tumor'/exp OR 'esophagus tumor':ab,ti OR 'neoplasm, esophageal':ab,ti OR 'esophagus neoplasm':ab,ti OR 'esophagus neoplasms':ab,ti OR 'neoplasm, esophagus':ab,ti OR 'neoplasms, esophagus':ab,ti OR 'neoplasms, esophageal':ab,ti OR 'cancer of esophagus':ab,ti OR 'cancer of the esophagus':ab,ti OR 'esophagus cancer':ab,ti OR 'cancer, esophagus':ab,ti OR 'cancers, esophagus':ab,ti OR 'esophagus cancers':ab,ti OR 'esophageal cancer':ab,ti OR 'cancer, esophageal':ab,ti OR 'cancers, esophageal':ab,ti OR 'esophageal cancers':ab,ti) AND ('immune checkpoint inhibitor'/exp OR 'checkpoint inhibitors, immune':ab,ti OR 'immune checkpoint inhibitor':ab,ti OR 'checkpoint inhibitor, immune':ab,ti OR 'immune checkpoint blockers':ab,ti OR 'checkpoint blockers, immune':ab,ti OR 'immune checkpoint blockade':ab,ti OR 'checkpoint blockade, immune':ab,ti OR 'immune checkpoint inhibition':ab,ti OR 'checkpoint inhibition, immune':ab,ti OR 'pd-l1 inhibitors':ab,ti OR 'pd l1 inhibitors':ab,ti OR 'pd-l1 inhibitor':ab,ti OR 'pd l1 inhibitor':ab,ti OR 'programmed death-ligand 1 inhibitors':ab,ti OR 'programmed death ligand 1 inhibitors':ab,ti OR 'ctla-4 inhibitors':ab,ti OR 'ctla 4 inhibitors':ab,ti OR 'ctla-4 inhibitor':ab,ti OR 'ctla 4 inhibitor':ab,ti OR 'cytotoxic t-lymphocyte-associated protein 4 inhibitors':ab,ti OR 'cytotoxic t lymphocyte associated protein 4 inhibitors':ab,ti OR 'pd 1 inhibitors':ab,ti OR 'pd-1 inhibitor':ab,ti OR 'inhibitor, pd-1':ab,ti OR 'programmed cell death protein 1 inhibitor':ab,ti OR 'pd-1-pd-l1 blockader':ab,ti OR 'pd 1 protein':ab,ti OR 'pd 1 receptor':ab,ti OR 'cd279 antigen':ab,ti OR 'pd1 receptor':ab,ti OR 'programmed cell death protein 1':ab,ti OR 'programmed cell death 1 protein':ab,ti OR 'carrelizumab':ab,ti OR 'opdivo':ab,ti OR 'ono-4538':ab,ti OR 'ono-45388':ab,ti OR 'bms-936558':ab,ti OR 'sch-900475':ab,ti OR 'mk-3475':ab,ti OR 'lambrolizumab':ab,ti OR 'ibi 308':ab,ti OR 'sintilimab':ab,ti OR 'tislelizumab':ab,ti OR 'bgb-a317':ab,ti OR 'nivolumab':ab,ti OR 'keytruda':ab,ti OR 'atezolizumab':ab,ti OR 'tecentriq':ab,ti OR 'imfinzi':ab,ti OR 'durvalumab':ab,ti OR 'shr-1210':ab,ti OR 'camrelizumab':ab,ti OR 'bavencio':ab,ti OR 'avelumab':ab,ti OR 'toripalimab':ab,ti OR 'cemiplimab':ab,ti)

Mapped termsn/a

**2.维普：119**

检索条件： (((((((((((题名或关键词=食管癌 OR 题名或关键词=carcinoma of esophagus) OR 题名或关键词=caricinoma of esophagus) OR 题名或关键词=esophageal cancer) OR 题名或关键词=esophageal carcinoma) OR 题名或关键词=esophagectomy) OR 题名或关键词=esophagus cancer) OR 题名或关键词=esophagus carcinoma) OR 题名或关键词=食道癌) AND ((((((((((((题名或关键词=免疫检查点抑制剂 OR 题名或关键词=卡瑞利珠单抗) OR 题名或关键词=纳武力尤单抗) OR 题名或关键词=帕博丽珠单抗) OR 题名或关键词=特瑞普利单抗) OR 题名或关键词=信迪利单抗) OR 题名或关键词=替雷利珠单抗) OR 题名或关键词=免疫治疗) OR 题名或关键词=immune therapy) OR 题名或关键词=immunization therapy) OR 题名或关键词=immunotherapeutic) OR 题名或关键词=immunotherapy) OR 题名或关键词=immunotherapy treatment))))

**3.知网：805**

[(主题=食管癌 + 食道癌) AND (主题=免疫治疗 + 免疫检查点抑制剂 + 卡瑞利珠单抗 + 纳武力尤单抗 + 帕博丽珠单抗 + 特瑞普利单抗 + 信迪利单抗 + 替雷利珠单抗 )](http://kns-cnki-net-s--bjmu.bjmu.toyss.me:8001/KNS8/AdvSearch?id=126&dbcode=SCDB&searchtype=gradeSearch&ishistory=1" \o "(主题=食管癌 + 食道癌) AND (主题=免疫治疗 + 免疫抑制剂 + 卡瑞利珠单抗 + 纳武力尤单抗 + 帕博丽珠单抗 + 特瑞普利单抗 + 信迪利单抗 + 替雷利珠单抗 )" \t "/Users/xinyu/Documents\\x/_blank)

**4.Corchane:427**

ID Search Hits

#1 (Esophageal Neoplasms):ti,ab,kw OR (esophagus tumor):ti,ab,kw OR (Neoplasm, Esophageal):ti,ab,kw OR (Esophagus Neoplasm):ti,ab,kw OR (Esophagus Neoplasms):ti,ab,kw (Word variations have been searched) 3082

#2 MeSH descriptor: [Esophageal Neoplasms] explode all trees 1688

#3 (Neoplasm, Esophagus):ti,ab,kw OR (Neoplasms, Esophagus):ti,ab,kw OR (Neoplasms, Esophageal):ti,ab,kw OR (Cancer of Esophagus):ti,ab,kw OR (Cancer of the Esophagus):ti,ab,kw (Word variations have been searched) 4179

#4 (Esophagus Cancer):ti,ab,kw OR (Cancer, Esophagus):ti,ab,kw OR (Cancers, Esophagus):ti,ab,kw OR (Esophagus Cancers):ti,ab,kw OR (Esophageal Cancer):ti,ab,kw (Word variations have been searched) 4760

#5 (Cancers, Esophageal):ti,ab,kw OR (Cancer, Esophageal):ti,ab,kw OR (Esophageal Cancers):ti,ab,kw (Word variations have been searched) 3832

#6 #1 or #2 or #3 or #4 or #5 5542

#7 MeSH descriptor: [Immune Checkpoint Inhibitors] explode all trees 26

#8 (Checkpoint Inhibitors, Immune):ti,ab,kw OR (Immune Checkpoint Inhibitor):ti,ab,kw OR (Immune Checkpoint Inhibitors):ti,ab,kw OR (Checkpoint Inhibitor, Immune):ti,ab,kw OR (Immune Checkpoint Blockers):ti,ab,kw (Word variations have been searched) 1115

#9 (Checkpoint Blockers, Immune):ti,ab,kw OR (Immune Checkpoint Blockade):ti,ab,kw OR (Checkpoint Blockade, Immune):ti,ab,kw OR (Immune Checkpoint Inhibition):ti,ab,kw OR (Checkpoint Inhibition, Immune):ti,ab,kw (Word variations have been searched) 476

#10 (PD-L1 Inhibitors):ti,ab,kw OR (PD L1 Inhibitors):ti,ab,kw OR (PD-L1 Inhibitor):ti,ab,kw OR (PD L1 Inhibitor):ti,ab,kw OR (Programmed Death-Ligand 1 Inhibitors):ti,ab,kw (Word variations have been searched) 962

#11 (Programmed Death Ligand 1 Inhibitors):ti,ab,kw OR (CTLA-4 Inhibitors):ti,ab,kw OR (CTLA 4 Inhibitors):ti,ab,kw OR (CTLA-4 Inhibitor):ti,ab,kw OR (CTLA 4 Inhibitor):ti,ab,kw (Word variations have been searched) 407

#12 (Cytotoxic T-Lymphocyte-Associated Protein 4 Inhibitors):ti,ab,kw OR (Cytotoxic T Lymphocyte Associated Protein 4 Inhibitors):ti,ab,kw OR (Cytotoxic T-Lymphocyte-Associated Protein 4 Inhibitor):ti,ab,kw OR (Cytotoxic T Lymphocyte Associated Protein 4 Inhibitor):ti,ab,kw OR (PD-1 Inhibitors):ti,ab,kw (Word variations have been searched) 543

#13 (PD 1 Inhibitors):ti,ab,kw OR (PD-1 Inhibitor):ti,ab,kw OR (Inhibitor, PD-1):ti,ab,kw OR (PD 1 Inhibitor):ti,ab,kw OR (Programmed Cell Death Protein 1 Inhibitor):ti,ab,kw (Word variations have been searched) 5210

#14 (PD 1 Protein):ti,ab,kw OR (PD-1 Receptor):ti,ab,kw OR (PD 1 Receptor):ti,ab,kw OR (Receptor, PD-1):ti,ab,kw OR (Antigens, CD279):ti,ab,kw (Word variations have been searched) 6525

#15 (CD279 Antigens):ti,ab,kw OR (CD279 Antigen):ti,ab,kw OR (Antigen, CD279):ti,ab,kw OR (PD1 Receptor):ti,ab,kw OR (Receptor, PD1):ti,ab,kw (Word variations have been searched) 73

#16 (Programmed Cell Death Protein 1):ti,ab,kw OR (Programmed Cell Death 1 Protein):ti,ab,kw OR (carrelizumab):ti,ab,kw OR (SHR-1210):ti,ab,kw OR (Opdivo):ti,ab,kw (Word variations have been searched) 595

#17 (ONO-4538):ti,ab,kw OR (BMS-936558):ti,ab,kw OR (MDX-1106):ti,ab,kw OR (SCH-900475):ti,ab,kw OR (Keytruda):ti,ab,kw (Word variations have been searched) 288

#18 (MK-3475):ti,ab,kw OR (lambrolizumab):ti,ab,kw OR (toripalimab):ti,ab,kw OR (IBI 308):ti,ab,kw OR (sintilimab):ti,ab,kw (Word variations have been searched) 454

#19 (tislelizumab):ti,ab,kw OR (BGB-A317):ti,ab,kw OR (Nivolumab):ti,ab,kw OR (pembrolizumab):ti,ab,kw OR (keytruda):ti,ab,kw (Word variations have been searched) 3790

#20 (atezolizumab):ti,ab,kw OR (tecentriq):ti,ab,kw OR (sintilimab):ti,ab,kw OR (imfinzi):ti,ab,kw OR (durvalumab):ti,ab,kw (Word variations have been searched) 1645

#21 (SHR-1210):ti,ab,kw OR (camrelizumab):ti,ab,kw OR (bavencio):ti,ab,kw OR (avelumab):ti,ab,kw OR (toripalimab):ti,ab,kw (Word variations have been searched) 415

#22 (cemiplimab):ti,ab,kw (Word variations have been searched) 49

#23 #7 or #8 or #9 or #10 or #11 or #12 or #13 #14 or #15 or #16 or #17 or #18 or #19 or #20 or #21 or #22 7951

#24 (Programmed Cell Death Protein 1 Inhibitors):ti,ab,kw (Word variations have been searched) 128

#25 (PD 1 PD L1 Blockade):ti,ab,kw (Word variations have been searched) 283

#26 (PD-1 Protein):ti,ab,kw (Word variations have been searched) 541

#27 #23 or #24 or #25 or #26 8025

#28 #6 and #27 215

5.生物医学：313

("免疫治疗"[常用字段] OR ("免疫检查点抑制剂"[常用字段] OR "免疫检查点抑制剂"[主题词]) OR "卡瑞利珠单抗"[常用字段] OR "纳武力尤单抗"[常用字段] OR "帕博丽珠单抗"[常用字段] OR "特瑞普利单抗"[常用字段] OR "信迪利单抗"[常用字段] OR "替雷利珠单抗"[常用字段]) AND (("食管癌"[常用字段] OR "食管肿瘤"[常用字段] OR "食道癌"[常用字段] OR "食管肿瘤"[主题词]) OR ("食道癌"[常用字段] OR "食管肿瘤"[常用字段] OR "食管癌"[常用字段] OR "食管肿瘤"[主题词]))

6.Pubmed：447

("Esophageal Neoplasms"[MeSH Terms]OR("neoplasm esophageal"[Title/Abstract]OR"esophagus tumor"[Title/Abstract]OR"esophagus neoplasm"[Title/Abstract]OR"esophagus neoplasms"[Title/Abstract]OR"neoplasm esophagus"[Title/Abstract]OR"neoplasms esophagus"[Title/Abstract]OR"neoplasms esophageal"[Title/Abstract]OR"cancer of esophagus"[Title/Abstract]OR"cancer of the esophagus"[Title/Abstract]OR"esophagus cancer"[Title/Abstract]OR"cancer esophagus"[Title/Abstract]OR"cancers esophagus"[Title/Abstract]OR"esophagus cancers"[Title/Abstract]OR"esophageal cancer"[Title/Abstract]OR"cancer esophageal"[Title/Abstract]OR"cancers esophageal"[Title/Abstract]OR"esophageal cancers"[Title/Abstract]))AND("immune checkpoint inhibitors"[Title/Abstract]OR"checkpoint inhibitors immune"[Title/Abstract]OR"immune checkpoint inhibitor"[Title/Abstract]OR"checkpoint inhibitor immune"[Title/Abstract]OR"immune checkpoint blockers"[Title/Abstract]OR"checkpoint blockers immune"[Title/Abstract]OR"immune checkpoint blockade"[Title/Abstract]OR"checkpoint blockade immune"[Title/Abstract]OR"immune checkpoint inhibition"[Title/Abstract]OR"checkpoint inhibition immune"[Title/Abstract]OR"pd l1 inhibitors"[Title/Abstract]OR"pd l1 inhibitors"[Title/Abstract]OR"pd l1 inhibitor"[Title/Abstract]OR"pd l1 inhibitor"[Title/Abstract]OR"programmed death ligand 1 inhibitors"[Title/Abstract]OR"programmed death ligand 1 inhibitors"[Title/Abstract]OR"ctla 4 inhibitors"[Title/Abstract]OR"ctla 4 inhibitors"[Title/Abstract]OR"ctla 4 inhibitor"[Title/Abstract]OR"ctla 4 inhibitor"[Title/Abstract]OR"cytotoxic t lymphocyte associated protein 4 inhibitors"[Title/Abstract]OR"cytotoxic t lymphocyte associated protein 4 inhibitors"[Title/Abstract]OR"cytotoxic t lymphocyte associated protein 4 inhibitor"[Title/Abstract]OR"cytotoxic t lymphocyte associated protein 4 inhibitor"[Title/Abstract]OR"pd 1 inhibitors"[Title/Abstract]OR"pd 1 inhibitors"[Title/Abstract]OR"pd 1 inhibitor"[Title/Abstract]OR"inhibitor pd 1"[Title/Abstract]OR"pd 1 inhibitor"[Title/Abstract]OR"programmed cell death protein 1 inhibitor"[Title/Abstract]OR"programmed cell death protein 1 inhibitors"[Title/Abstract]OR"pd 1 pd l1 blockade"[Title/Abstract]OR"blockade pd 1 pd l1"[Title/Abstract]OR"pd 1 pd l1 blockade"[Title/Abstract]OR"pd 1 protein"[Title/Abstract]OR"pd 1 protein"[Title/Abstract]OR"pd 1 receptor"[Title/Abstract]OR"pd 1 receptor"[Title/Abstract]OR"receptor pd 1"[Title/Abstract]OR"antigens cd279"[Title/Abstract]OR("CD279"[All Fields]AND"Antigens"[Title/Abstract])OR("CD279"[All Fields]AND"Antigen"[Title/Abstract])OR(("antigen s"[All Fields]OR"antigene"[All Fields]OR"antigenes"[All Fields]OR"antigenic"[All Fields]OR"antigenically"[All Fields]OR"antigenicities"[All Fields]OR"antigenicity"[All Fields]OR"antigenized"[All Fields]OR"Antigens"[MeSH Terms]OR"Antigens"[All Fields]OR"Antigen"[All Fields])AND"CD279"[Title/Abstract])OR"pd1 receptor"[Title/Abstract]OR"receptor pd1"[Title/Abstract]OR"programmed cell death protein 1"[Title/Abstract]OR"programmed cell death 1 protein"[Title/Abstract]OR("carrelizumab"[Title/Abstract]OR"SHR-1210"[Title/Abstract]OR"Opdivo"[Title/Abstract]OR"ONO-4538"[Title/Abstract]OR"MDX-1106"[Title/Abstract]OR"BMS-936558"[Title/Abstract]OR"SCH-900475"[Title/Abstract]OR"Keytruda"[Title/Abstract]OR"MK-3475"[Title/Abstract]OR"lambrolizumab"[Title/Abstract]OR"toripalimab"[Title/Abstract]OR("IBI"[All Fields]AND"308"[Title/Abstract])OR"sintilimab"[Title/Abstract]OR"tislelizumab"[Title/Abstract]OR"BGB-A317"[Title/Abstract]OR"Nivolumab"[Title/Abstract]OR"pembrolizumab"[Title/Abstract]OR"Keytruda"[Title/Abstract]OR"atezolizumab"[Title/Abstract]OR"tecentriq"[Title/Abstract]OR"sintilimab"[Title/Abstract]OR"imfinzi"[Title/Abstract]OR"durvalumab"[Title/Abstract]OR"SHR-1210"[Title/Abstract]OR"camrelizumab"[Title/Abstract]OR"bavencio"[Title/Abstract]OR"avelumab"[Title/Abstract]OR"toripalimab"[Title/Abstract]OR"cemiplimab"[Title/Abstract]))

**7.Web of science：1085**

**#1.TOPIC:**(Esophageal Neoplasms)*OR* **TOPIC:**(esophagus tumor)*OR* **TOPIC:**(Neoplasm,Esophageal)*OR* **TOPIC:**(Esophagus Neoplasm)*OR* **TOPIC:**(Esophagus Neoplasms)*OR* **TOPIC:**(Neoplasm,Esophagus)*OR* **TOPIC:**(Neoplasms,Esophagus)*OR* **TOPIC:**(Neoplasms,Esophageal)*OR* **TOPIC:**(Cancer of Esophagus)*OR* **TOPIC:**(Cancer of the Esophagus)*OR* **TOPIC:**(Esophagus Cancer)*OR* **TOPIC:**(Cancer,Esophagus)*OR* **TOPIC:**(Cancers,Esophagus)*OR* **TOPIC:**(Esophagus Cancers)*OR* **TOPIC:**(Esophageal Cancer)*OR* **TOPIC:**(Cancer,Esophageal)*OR* **TOPIC:**(Cancers,Esophageal)*OR* **TOPIC:**(Esophageal Cancers)

*Indexes=SCI-EXPANDED,SSCI,A&HCI,CPCI-S,CPCI-SSH,ESCI,CCR-EXPANDED,IC Timespan=All years*

***#2* TOPIC:**(Immune Checkpoint Inhibitors)*OR* **TOPIC:**(Checkpoint Inhibitors,Immune)*OR* **TOPIC:**(Immune Checkpoint Inhibitor)*OR* **TOPIC:**(Checkpoint Inhibitor,Immune)*OR* **TOPIC:**(Immune Checkpoint Blockers)*OR* **TOPIC:**(Checkpoint Blockers,Immune)*OR* **TOPIC:**(Immune Checkpoint Blockade)*OR* **TOPIC:**(Checkpoint Blockade,Immune)*OR* **TOPIC:**(Immune Checkpoint Inhibition)*OR* **TOPIC:**(Checkpoint Inhibition,Immune)*OR* **TOPIC:**(PD-L1 Inhibitors)*OR* **TOPIC:**(PD L1 Inhibitors)*OR* **TOPIC:**(PD-L1 Inhibitor)*OR* **TOPIC:**(PD L1 Inhibitor)*OR* **TOPIC:**(Programmed Death-Ligand 1 Inhibitors)*OR* **TOPIC:**(Programmed Death Ligand 1 Inhibitors)*OR* **TOPIC:**(CTLA-4 Inhibitors)*OR* **TOPIC:**(CTLA 4 Inhibitors)*OR* **TOPIC:**(CTLA-4 Inhibitor)*OR* **TOPIC:**(CTLA 4 Inhibitor)*OR* **TOPIC:**(Cytotoxic T-Lymphocyte-Associated Protein 4 Inhibitors)*OR* **TOPIC:**(Cytotoxic T Lymphocyte Associated Protein 4 Inhibitors)*OR* **TOPIC:**(Cytotoxic T-Lymphocyte-Associated Protein 4 Inhibitor)*OR* **TOPIC:**(Cytotoxic T Lymphocyte Associated Protein 4 Inhibitor)*OR* **TOPIC:**(PD-1 Inhibitors)

*Indexes=SCI-EXPANDED,SSCI,A&HCI,CPCI-S,CPCI-SSH,ESCI,CCR-EXPANDED,IC Timespan=All years*

***#3*** TOPIC:(PD 1 Inhibitors)OR TOPIC:(PD-1 Inhibitor)OR TOPIC:(Inhibitor,PD-1)OR TOPIC:(PD 1 Inhibitor)OR TOPIC:(Programmed Cell Death Protein 1 Inhibitor)OR TOPIC:(Programmed Cell Death Protein 1 Inhibitors)OR TOPIC:(PD-1-PD-L1 Blockade)OR TOPIC:(Blockade,PD-1-PD-L1)OR TOPIC:(PD 1 PD L1 Blockade)OR TOPIC:(PD-1 Protein)OR TOPIC:(PD 1 Protein)OR TOPIC:(PD-1 Receptor)OR TOPIC:(PD 1 Receptor)OR TOPIC:(Receptor,PD-1)OR TOPIC:(Antigens,CD279)OR TOPIC:(CD279 Antigens)OR TOPIC:(CD279 Antigen)OR TOPIC:(Antigen,CD279)OR TOPIC:(PD1 Receptor)OR TOPIC:(Receptor,PD1)OR TOPIC:(Programmed Cell Death Protein 1)OR TOPIC:(Programmed Cell Death 1 Protein)OR TOPIC:(carrelizumab)OR TOPIC:(SHR-1210)OR TOPIC:(Opdivo)

Indexes=SCI-EXPANDED,SSCI,A&HCI,CPCI-S,CPCI-SSH,ESCI,CCR-EXPANDED,IC Timespan=All years

**#4TOPIC:**(ONO-4538)*OR* **TOPIC:**(MDX-1106)*OR* **TOPIC:**(BMS-936558)*OR* **TOPIC:**(SCH-900475)*OR* **TOPIC:**(Keytruda)*OR* **TOPIC:**(MK-3475)*OR* **TOPIC:**(lambrolizumab)*OR* **TOPIC:**(toripalimab)*OR* **TOPIC:**(IBI 308)*OR* **TOPIC:**(sintilimab)*OR* **TOPIC:**(tislelizumab)*OR* **TOPIC:**(BGB-A317)*OR* **TOPIC:**(Nivolumab)*OR* **TOPIC:**(pembrolizumab)*OR* **TOPIC:**(keytruda)*OR* **TOPIC:**(atezolizumab)*OR* **TOPIC:**(tecentriq)*OR* **TOPIC:**(sintilimab)*OR* **TOPIC:**(imfinzi)*OR* **TOPIC:**(durvalumab)*OR* **TOPIC:**(SHR-1210)*OR* **TOPIC:**(camrelizumab)*OR* **TOPIC:**(bavencio)*OR* **TOPIC:**(avelumab)*OR* **TOPIC:**(toripalimab)

*Indexes=SCI-EXPANDED,SSCI,A&HCI,CPCI-S,CPCI-SSH,ESCI,CCR-EXPANDED,IC Timespan=All years*

***#5*TOPIC:**(cemiplimab)

*Indexes=SCI-EXPANDED,SSCI,A&HCI,CPCI-S,CPCI-SSH,ESCI,CCR-EXPANDED,IC Timespan=All years*

***#6***#5 OR#4 OR#3 OR#2

*Indexes=SCI-EXPANDED,SSCI,A&HCI,CPCI-S,CPCI-SSH,ESCI,CCR-EXPANDED,IC Timespan=All years*

***#7***#6 AND#1

*Indexes=SCI-EXPANDED,SSCI,A&HCI,CPCI-S,CPCI-SSH,ESCI,CCR-EXPANDED,IC Timespan=All years*

1. **万方：1001**

主题:(食管癌 or 食道癌) and 主题:(免疫检查点抑制剂 or 免疫治疗 or 免疫靶点抑制剂 or 卡瑞利珠单抗 or 纳武力尤单抗 or 帕博丽珠单抗 or 特瑞普利单抗 or 信迪利单抗 or 替雷利珠单抗)
