# Supplementary material for: Comparative efficacy and toxicity of immune checkpoint inhibitors in combination with or without chemotherapy treatment for advanced esophageal squamous cell carcinoma: A systematic review and meta-analysis
Source: Front Oncol. 2022 Nov 24;12:958783. doi: 10.3389/fonc.2022.958783 (PMC9748809; doi:10.3389/fonc.2022.958783)
Supplement: Supplement File 2 — modified Jadad scale. [file Table_2.docx]

| Author | Was the study described as randomized? | Was the method of randomization appropriate? | Was the study described as blinded? | Was the method of blinding appropriate? | Was there a description of withdrawals and dropouts? | Was there a clear description of the inclusion/exclusion criteria? | Was the method used to assess adverse effects described？ | Was the method of statistical analysis described? | Modified Jadad Score |
| --- | --- | --- | --- | --- | --- | --- | --- | --- | --- |
| Huiyan Luo | 1 | 1 | 1 | 1 | 1 | 1 | 1 | 1 | 8 |
| Ian Chau | 1 | 1 | 1 | 0 | 0 | 1 | 1 | 1 | 6 |
| J. Ajani | 0 | 0 | 0 | 0 | 0 | 0 | 0 | 0 | 0 |
| Jianming Xu | 1 | 1 | 1 | 1 | 1 | 1 | 1 | 1 | 8 |
| Jing Huang | 1 | 1 | -1 | -1 | 1 | 1 | 1 | 1 | 4 |
| Jong-Mu Sun | 1 | 1 | 1 | 1 | 1 | 1 | 1 | 1 | 8 |
| Ken Kato | 1 | 1 | -1 | 0 | 1 | 1 | 1 | 1 | 5 |
| Lin Shen 2021 | 0 | 0 | 0 | 1 | 0 | 0 | 0 | 0 | 1 |
| Masanobu Takahashi | 1 | 1 | 1 | -1 | 1 | 1 | 1 | 1 | 6 |
| R.Xu | 0 | 0 | 0 | 1 | 0 | 0 | 0 | 0 | 1 |
| Takashi Kojima | 0 | 0 | -1 | 1 | 1 | 1 | 1 | 1 | 4 |
| Y.cao | 0 | 1 | 0 | 0 | 1 | 1 | 1 | 1 | 5 |
| Xiaochuan Liu | 1 | 1 | -1 | 0 | 1 | 1 | 1 | 1 | 5 |
| Zhihao Lu | 0 | 1 | 1 | 1 | 1 | 1 | 1 | 1 | 7 |
| Lin Shen 2022 | 0 | 0 | 0 | 0 | 0 | 0 | 0 | 0 | 0 |
| H.Yoon | 0 | 0 | 0 | 0 | 0 | 0 | 0 | 0 | 0 |
| Doki Y | 1 | 1 | 1 | 1 | 1 | 1 | 1 | 1 | 8 |

Quality Assessment of the Included Studies According to Modified Jadad score
