# Supplementary material for: Comparative efficacy and toxicity of immune checkpoint inhibitors in combination with or without chemotherapy treatment for advanced esophageal squamous cell carcinoma: A systematic review and meta-analysis
Source: Front Oncol. 2022 Nov 24;12:958783. doi: 10.3389/fonc.2022.958783 (PMC9748809; doi:10.3389/fonc.2022.958783)
Supplement: Supplement File 3 — Sensitivity analysis. [file Table_3.docx]

**sensitivity analysis**

1.OS

**1.1 First-line**

**1.2 Second-line**

1. 12 OS

**2.1 First-line**

2.2 Second-line

1. **Treatment-related adverse reactions**

3.1 First-line

3.2 Second-line

1. **Treatment-related adverse reactions of grade 3 or higher**

4.1 First-line

4.2 Second-line

1. DCR（**Disease control rate**）

5.1 First-line

5.2 Second-line

1. **ORR (Objective response rate)**

6.1 First-line

6.2 Second-line

1. **PFS (Progression-free survival)**

7.1 First-line

7.2 Second-line

1. **Weakness**

1. **Hypothyroidism**

1. **Diarrhoea**

1. **Anemia**

1. Rash

1. **Decreased appetites**
